# Three-Dimensional Reconstruction of Light Field Based on Phase Similarity

**DOI:** 10.3390/s21227734

**Published:** 2021-11-20

**Authors:** Wei Feng, Junhui Gao, Tong Qu, Shiqi Zhou, Daxing Zhao

**Affiliations:** Hubei Key Laboratory of Modern Manufacturing Quality Engineering, School of Mechanical Engineering, Hubei University of Technology, Wuhan 430068, China; junhuigao@hbut.edu.cn (J.G.); qutong@hbut.edu.cn (T.Q.); 102000031@hbut.edu.cn (S.Z.); zdx007@126.com (D.Z.)

**Keywords:** light field, phase information, 3D reconstruction, fringes projection

## Abstract

Light field imaging plays an increasingly important role in the field of three-dimensional (3D) reconstruction because of its ability to quickly obtain four-dimensional information (angle and space) of the scene. In this paper, a 3D reconstruction method of light field based on phase similarity is proposed to increase the accuracy of depth estimation and the scope of applicability of epipolar plane image (EPI). The calibration method of the light field camera was used to obtain the relationship between disparity and depth, and the projector calibration was removed to make the experimental procedure more flexible. Then, the disparity estimation algorithm based on phase similarity was designed to effectively improve the reliability and accuracy of disparity calculation, in which the phase information was used instead of the structure tensor, and the morphological processing method was used to denoise and optimize the disparity map. Finally, 3D reconstruction of the light field was realized by combining disparity information with the calibrated relationship. The experimental results showed that the reconstruction standard deviation of the two objects was 0.3179 mm and 0.3865 mm compared with the ground truth of the measured objects, respectively. Compared with the traditional EPI method, our method can not only make EPI perform well in a single scene or blurred texture situations but also maintain good reconstruction accuracy.

## 1. Introduction

Light field imaging as new imaging technology has become research hotspots. It is dedicated to simultaneously recording the direction and intensity information of light, and its characteristic ensures that the light field camera can obtain multiple viewing angles with a single exposure [1,2]. Therefore, light field imaging can be applied to the three-dimensional (3D) reconstruction fields, such as physical measurement [3,4], depth estimation [5,6], intelligent detection [7,8], and so on.

As is known to all that depth estimation is one of the most important research contents in 3D reconstruction [9]. At present, there are two main ways to estimate depth information by using light field data. One is the multi-view image matching, and the other is EPI based on structural information.

The multi-view image matching method requires all sub-aperture images to be matched to obtain the scene disparity. Yu et al. explored the geometry of light in three-dimensional space to improve the accuracy of stereo matching, but it did not perform well when the disparity of each pixel was small [10]. A new matching method based on principal component analysis was proposed to realize multi-view stereo reconstruction [11]. An occlusion prediction framework of light field images had been proven effective in the occlusion scene, and it was able to identify the obscured edges [12]. Tao et al. estimated the depth of the scene using both defocus and matching cues and then combined shadow cues to improve the ability of detail recovery [13,14]. However, it had led to increased calculation and poor real-time performance. In addition, a bidirectional reflectance distribution function invariant theory of spatial variation was deduced to restore 3D shape and estimate the depth of non-Lambert planes [15]. Although the information in the whole scene could be utilized perfectly by using multi-view images, it inevitably led to a large amount of calculation and time-consuming.

The other method is EPI based on structural information to obtain the slope of the line structure corresponding to the pixel points, where the slope corresponds to the disparity. The EPI based on structure tensor was used to calculate the depth of the scene, and the EPI lines were extracted to calculate its slope under the framework of total variation [16]. A method based on scene confidence was designed to improve the accuracy of EPI depth estimation and leveraged the coherence of light fields to achieve the goal of 3D reconstruction [17]. In addition, sheared EPI analysis was performed well where EPIs were transformed with several shear values before the structure tensor analysis to estimate accurate disparities even from non-dense light fields [18]. However, EPI methods based on structure tensor depend too much on the complexity of scene texture, and it is difficult to show good effect in the situation of a single texture scene. The above methods still need to be further optimized for regions with similar or missing textures.

3D reconstruction with structured light is regarded as the main measuring technology for its high accuracy and fast speed [19]. Sinusoidal fringes are projected onto the object surface, and the phase information modulated by the object surface will be obtained by the multi-frequency heterodyne method. In this case, there is a unique determination relationship between the phase value and the pixel in the same scene. The method used the structured light in the light field had realized the 3D measurement [20]. However, their work still used the camera and projector calibration technology, so the system calibration was more complicated due to the feature of the multi-view angle of the light field camera. Besides, the phase modulation degrees of two-step phase shift was not obvious, and it was difficult to achieve accurate 3D reconstruction. Consequently, we take the structured light fringe projection technique into the calculation of light field EPI to make sure that each pixel in the scene can be encoded with phase information, and the four-step shifted phase algorithm is used to make the phases independent of each other.

In this paper, a 3D reconstruction method of light field based on phase similarity is proposed to make EPI perform well in the single texture scene. The process of system calibration in the structured light method is eliminated, and there is no need to conduct secondary calibration for the projector. According to the model of light field imaging, the calibration for the light field camera based on Zhang’s calibration method is realized to obtain the relationship between disparity and depth. This relationship must lay the foundation for the 3D reconstruction of the light field. Then the deep estimation algorithm based on phase similarity is designed to effectively improve the reliability and accuracy of disparity calculation, where the phase information is used to replace the structure tensor. The morphological processing method is used to denoise and optimizes the disparity map to improve its accuracy. The 3D reconstruction of the light field can be realized by combining the disparity information based on phase similarity with the linear relationship obtained from calibration. Generally, our methods can not only make EPI perform well in a single scene or blurred texture situation but also maintain good reconstruction accuracy.

The rest of this paper is arranged as follows. Section 2 introduces the principle of our method and our hardware implement. Section 3 describes the imaging model of the light field camera and its calibration method. Section 4 presents the principle of light field 3D reconstruction based on phase similarity and explains it in detail. The experimental results will be shown in Section 5, and Section 6 summarizes the work and discusses further research.

## 2. Principles

### 2.1. Light Field Imaging and EPI Principle

**Figure 1:**
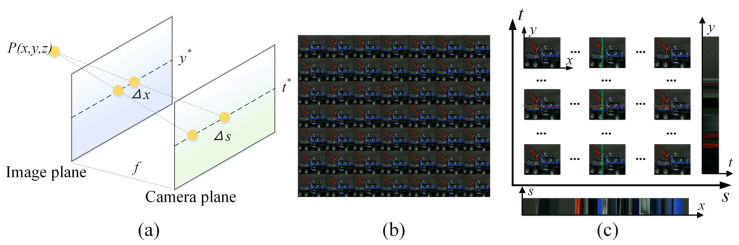
Light field imaging. (**a**) Light field EPI principle; (**b**) Multi-view images; (**c**) Epipolar plane image.

On the basis of the pinhole model of a traditional camera, the micro-lens array (MLA) is added between the main lens and the image sensor. The incident light is converged onto the MLA through the main lens, and then re-imaged onto the image sensor. Therefore, the light field camera can record the four-dimensional information of angle and space simultaneously. The accepted model of a light field camera is a geometric model based on a two-parallel plane [21]. Generally, light defined as a straight line passes through the main lens plane (*s*, *t*) and the micro-lens plane (*x*, *y*) to form a number of pixel points, where point *P* is projected onto two lines of two planes with fixed coordinates, as presented in Figure 1a. The main lens plane (*s*, *t*) provides the angular resolution of the scene, and the micro-lens plane (*x*, *y*) provides the spatial resolution of the scene, hence the light field can be represented as *L* (*s*, *t*, *x*, *y*).

The light field *L* (*s*, *t*, *x*, *y*) can be simply understood as a function of the light space. In particular, when the light space is limited to a two-dimensional plane, the light field can be represented as L_t__*, y*_ = *L* (*s*, t*, *x*, y*). Similarly, other restrictions can be defined in the same way. For example, L_s__*, t*_ is a sub-aperture image of a particular angle in the light field image. Figure 1b shows 7 × 7 sub-aperture images extracted from the 4D light field data, in which the size of each sub-aperture image is 512 pixels × 512 pixels. L_s*__, x*,_ and L_t*__, y*_ are referred to as EPI, and EPI can be understood as a horizontal or vertical two-dimensional slice of 4D light field data, as shown in Figure 1c.

Light field cameras contain multi-angle information and there is a linear relationship between the change of view and the projection coordinates on the EPI plane. The rate of change depends on the depth of the projected scene point, which is also called disparity. This feature makes EPI present a unique structure and ensures that a projection point is a straight line in EPI.

### 2.2. The Principle of 3D Reconstruction of Light Field Based on Phase Similarity

**Figure 2:**
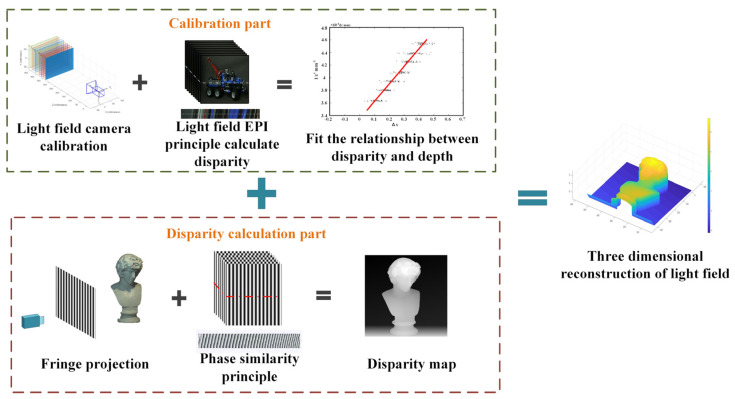
The principle of 3D reconstruction of light field based on phase similarity.

Our proposed 3D reconstruction method of light field based on phase similarity is mainly divided into two parts: the calibration part and the disparity calculation part, as shown in Figure 2.

The calibration part is to establish a linear relationship between disparity and depth. The linear relationship between disparity and depth can be deduced by analyzing the light field imaging model. Depth is obtained by Zhang’s calibration method by capturing the chessboard from different positions [22], and the disparity is obtained by using the EPI method to calculate the information of chessboard feature points. Finally, the Levenberg-Marquardt algorithm [23] is used to improve the reliability of the results.

The disparity calculation part is to uses the principle that phase presents similarity in EPI. Firstly, Sine fringes with different phase values are projected onto the surface, the multi-frequency heterodyne phase method is used to unwrap the phase-coded scenes. And then, the pixels of the middle row or column are selected as the target points in the EPI of the structured light field. The point with the highest similarity to the phase information of the target pixel is searched in other rows (or columns) and then those points will be fitted into a straight line. The slope of the line corresponds to the disparity. The disparity maps of the scene can be accurately acquired from the entire light field data in a similar way.

Based on the above, 3D reconstruction of the light field can be realized finally. It can be realized by combining the accurate disparity map based on phase similarity with the linear relationship deduced from the light field imaging model.

## 3. Calibration

In the traditional pinhole camera model, it is known by the Gauss imaging theorem:

(1) $$\frac{1}{u}+\frac{1}{v}=\frac{1}{f},$$

where *u* represents the distance between the scene point *P*_0_ and the main lens, *v* represents the distance between the imaging plane and the main lens, and *f* is the focal length of the main lens.

The traditional camera calibration is to calculate the corresponding relationship between the world coordinate and the imaging coordinate. Here, the calibration method of the light field camera is to obtain the mapping relationship between the disparity and depth.

**Figure 3:**
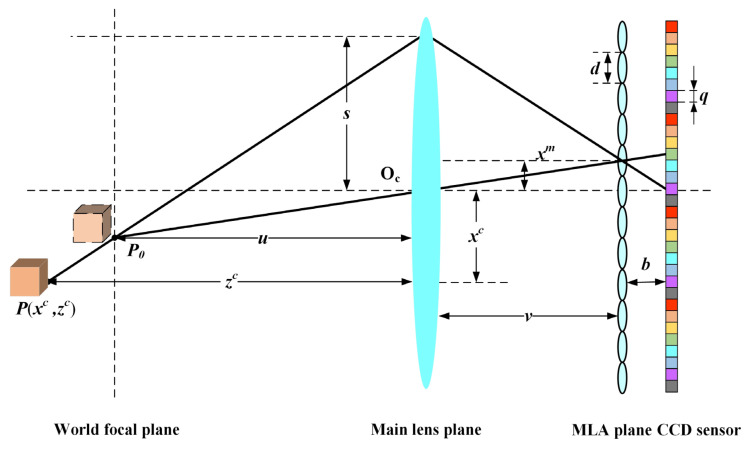
Imaging model of light field camera.

Figure 3 shows the light field imaging model [24], and the relationship between scene points *P* (*x^c^*, *z^c^*) and image points in MLA plan can be represented as:

(2) $$\frac{x^{m}}{v}=\frac{s-x^{c}}{z^{c}}-\frac{s}{u},$$

where *s* represents the distance from the main lens sub-aperture to the optical center *O_c_*, and *x^m^* is the distance from the MLA’s center to the position where the light passes through the MLA plane.

**Figure 4:**
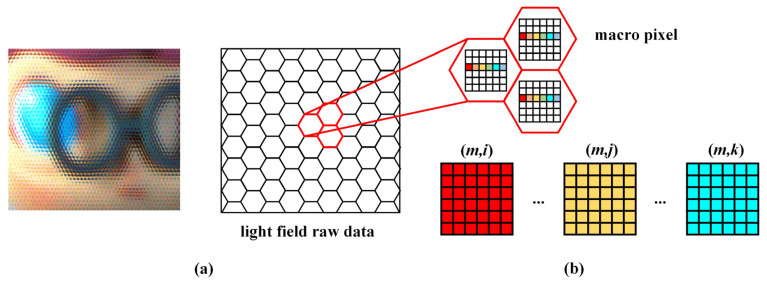
Sub-aperture imaging principle. (**a**) Light field image raw data; (**b**) Sub-aperture image extraction method.

The principle of sub-aperture imaging is shown in Figure 4. The hexagonal macro pixels in the light field raw data are shown in Figure 4a. Figure 4b shows the principle of sub-aperture image extraction in which the pixels in different macro pixels are arranged in order. Different from traditional images, the light field raw data consists of hexagonal macro pixels, and each macro pixel corresponds to an area in the image sensor that is covered by a micro lens. Sub-aperture images are extracted in a certain sequence from each macro pixel of the light field raw data. In general, (*m*, *n*) is used to represent the sub-aperture area on the main lens, and the distance *D* between the two adjacent sub-aperture areas can be expressed as:

(3) $$D=\frac{qv}{b},$$

where *q* is the size of the pixels on the image sensor, and *b* is the distance between the MLA and the image sensor. The relationship between *s* and the index of pixels *m* can be expressed as:

(4) $$s=\frac{qv(m-m_{0})}{b}=D(m-m_{0})$$

Equation (5) represents two adjacent sub-apertures, then Equation (2) is rewritten as Equation (6):

(5) $$\{\begin{array}{c} s_{l}=D(m_{l}-m_{0})\\ s_{l+1}=D(m_{l+1}-m_{0}) \end{array},$$

(6) $$\{\begin{array}{c} \frac{x^{m_{1}}}{v}=\frac{D(m_{l}-m_{0})-x^{c}}{z^{c}}-\frac{s_{l}}{u}\\ \frac{x^{m_{2}}}{v}=\frac{D(m_{l+1}-m_{0})-x^{c}}{z^{c}}-\frac{s_{l+1}}{u} \end{array},$$

Subtracting the top equation from the bottom one in Equation (6) yields the expression:

(7) $$\frac{1}{z^{c}}=\frac{1}{u}+\frac{x^{m_{2}}-x^{m_{1}}}{Dv},$$

(8) $$\Delta x=(x^{m_{2}}-x^{m_{1}})/d,$$

where *d* is the distance between the center of the adjacent micro-lens. Δ*x* represents the disparity values between the two adjacent sub-aperture views of scene point *P*, and it can be calculated from EPI. It should be noted that Δ*x* is independent of *l*, which means the light field disparity values are the same in any two adjacent sub-aperture images. It describes a linear relationship between the reciprocal of scene depth 1/*z^c^* and disparity values Δ*x* from Equation (8). According to the disparity calculation relationship, Equation (7) can be rewritten as Equation (9).

(9) $$\frac{1}{z^{c}}=\frac{1}{u}+\frac{bd}{qv^{2}}(\Delta x),$$

Therefore, the central sub-aperture image captured by the light field camera is equivalent to the image captured by the traditional camera, so the main lens parameters of the light field camera can be calibrated by taking a chessboard with different positions based on Zhang’s calibration method.

Finally, a non-linear optimization algorithm named Levenberg-Marquardt is used to minimize the optimization of the obtained linear equation. It is insensitive to over-parameterized and can effectively deal with redundant parameters. Therefore, LM minimization can be used to optimize the fitted linear equation, which can effectively improve the reliability of the linear relationship.

The system calibration in the structured light method is eliminated, and there is no need to conduct secondary calibration for the projector. Instead, the relationship between disparity and depth can be obtained through camera calibration, which can quickly realize 3D reconstruction.

## 4. Disparity Calculation

In the EPI calculation of the light field, each pixel is obtained by calculating the image gradient and structure tensor, and then the line slope corresponds to the disparity of each pixel in the sub-aperture image. However, when the measured object encounters texture loss or similar regions, the line structure in the EPI may no longer be clear, which makes it difficult to directly calculate the slope of the line for each pixel in the scene.

The light field sub-aperture image can be regarded as viewing objects from different angles [25]. According to the phase measurement profilometry (PMP), the phase values of the same target point modulated in different angles should be the same, which makes the phase information present similarity in the corresponding straight-line direction of EPI. Therefore, the EPI method based on phase information can replace the structural tensor method.

**Figure 5:**
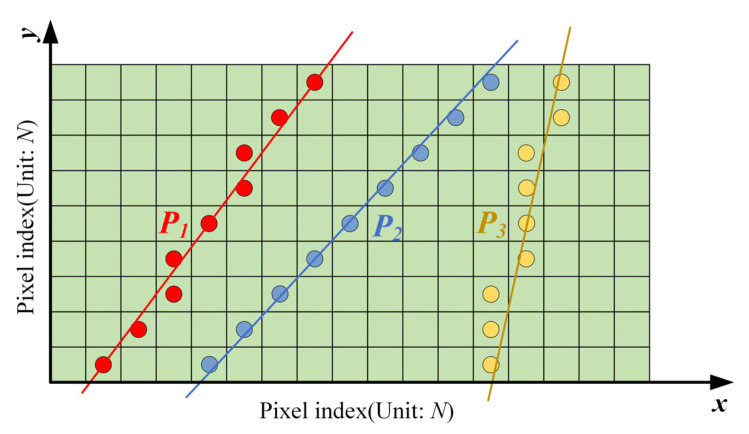
Principle of phase similarity.

For example, the positions of points in other rows or columns should be recorded to determine the slope of a single pixel (s^*^, x^*^) in an EPI image (*s*, *x*) of a central-view structured light field, and those points have the highest similarity from the phase value of the target point in the light field slice. The slope of the line at the points (s^*^, x^*^) can be obtained by linear fitting of these points, as shown in Figure 5. Each point can similarly get the corresponding slope, and then the disparity map can be obtained. The detailed implementation steps are as follows:

Step 1: Project sinusoidal fringes onto the object surface. The phase information is combined with the shifted phase method to encode the object.

Step 2: Acquisition of fringe images. The frequency of the sinusoidal fringe should be selected appropriately due to the limitation of the resolution and frame rate of the light field camera, which is used to capture the sinusoidal fringes.

Step 3: Phase unwrapping. The multifrequency heterodyne method is used to phase unwrap 4D light field data.

Step 4: Searching and recording the highest phase similarity. In the light field, EPI is based on phase information, and those point positions which have the highest phase value similarity to the target point are recorded in other rows (or columns).

Step 5: Fitting straight line. According to the position index of these points, the linear fitting method is used to fit them into a straight line.

Step 6: Disparity calculation based on phase similarity. The line slope is calculated and then the disparity can be obtained by taking the inverse of the slope. The accurate disparity map can be acquired by traversing the entire light field data based on phase information encoding.

Our proposed method can effectively improve the complexity of the operation. It is worth noting that although the calibration part of the system is saved, it is necessary to filter and denoise the obtained disparity map. The opening operation in morphological processing is composed of erode and dilate operation, and it can effectively eliminate isolated small noise points in the image and smooth the object edges without changing the object shape [26]. Here, the morphological operation is used to process the disparity information to eliminate the small noise points and small black areas separated from the object. The clear edge contour of the object is retained to effectively denoise, which provides a guarantee to realize the 3D reconstruction of the light field.

## 5. Hardware and Experiments

### 5.1. Hardware Implementation

**Figure 6:**
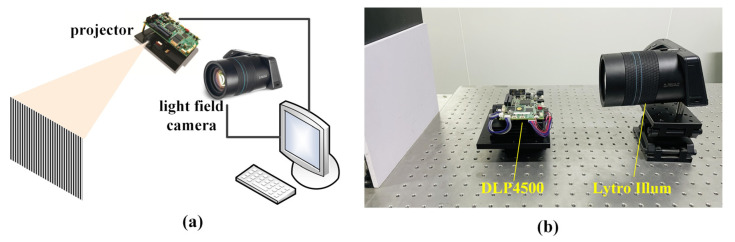
3D reconstruction system based on a light field. (**a**) Schematic diagram; (**b**) Hardware Implementations.

According to the principle of light field imaging and EPI, we have designed and built a 3D reconstruction system based on a structured light field, and it consisted of a light field camera Lytro Illum (San Francisco, CA, USA) and Light Crafter 4500, as shown in Figure 6. The light field camera had an angular resolution of 15 × 15 and the spatial resolution of 625 pixels × 433 pixels; Light Crafter 4500 was made by Texas Instruments with 1140 pixels × 912 pixels resolution. Computer configuration included Windows 10 (64 bit); Intel (R) Core (TM) i9-9900 K CPU @ 3.60 GHz. LFToolbox designed by Stanford was exploited to decode 4D light field data *L* (*s*, *t*, *x*, *y*) and obtain the multi-view information of the light field [27].

### 5.2. Experimental Results

**Figure 7:**
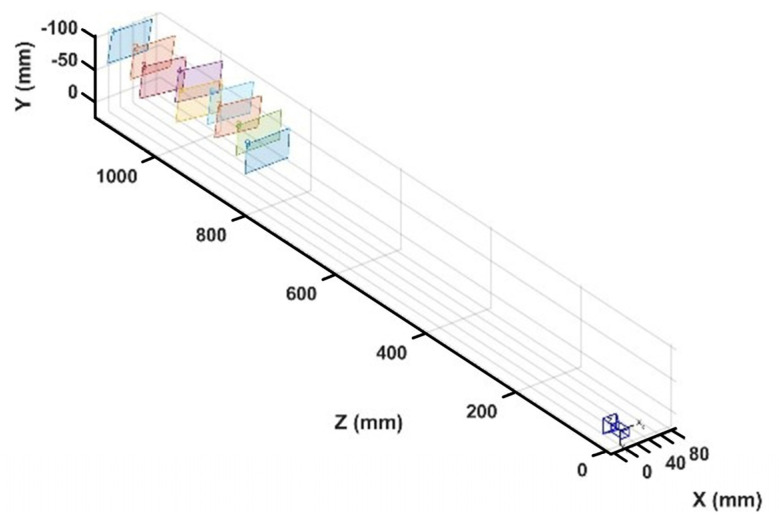
Calibration posture results of light field cameras.

**Figure 8:**
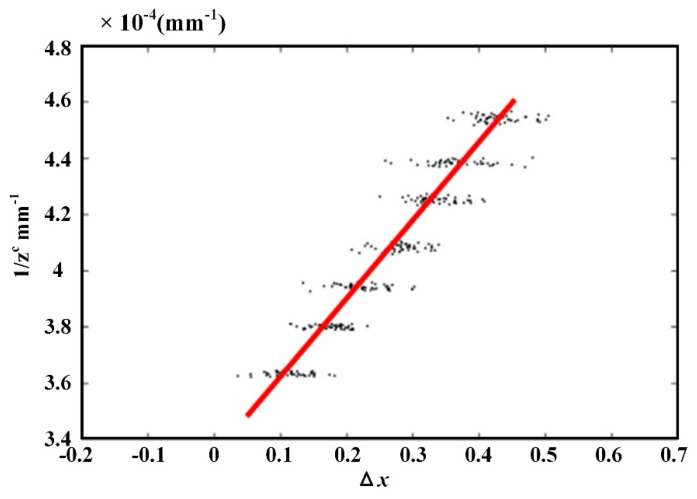
Linear fitting results of disparity and depth.

In our experiment, the 3D reconstruction system based on the light field had been shown in Figure 6. The purpose of the calibration was to fit the mapping relationship between disparity and depth according to the disparity and depth values at feature points of the chessboard. The depth information of each checkerboard corner is obtained based on Zhang’s calibration method by taking different positions of the checkerboard, and then the disparity values of the checkerboard corner are calculated based on the EPI principle. Nine kinds of postures were collected from a chessboard with a corner size of 24.5 × 24.5 mm and a corner number of 9 × 7. The light field camera and the posture distribution of the chessboard were shown in Figure 7. The data showed that the center distance *d* of two adjacent micro-lenses was 0.01732 mm and the size *q* of a single pixel on the image sensor was 0.0014 mm. To achieve the purpose of calibration, EPI could be obtained in the decoded light field data, the disparity and depth values at each feature point were calculated, and the mapping relationship between disparity and depth was fitted as shown in Figure 8. The relationship between disparity and depth was obtained as follows:

(10) $$1/z_{c}=3.385\times 10^{-4}+3.439\times 10^{-4}\Delta x,$$

**Figure 9:**
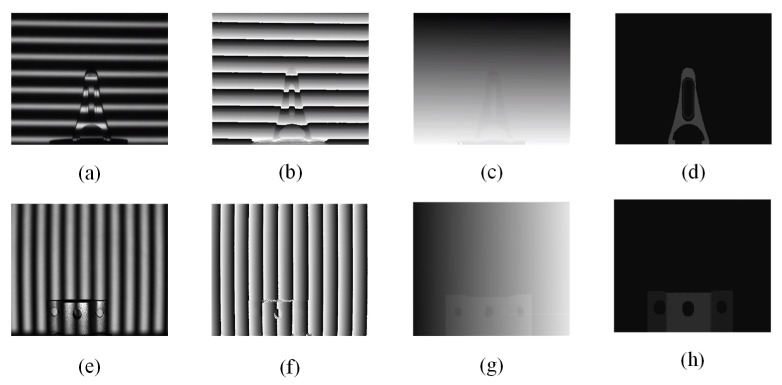
The two objects in the figure were projected by horizontal and vertical fringe. (**a**,**e**) were fringe images, (**b**,**f**) were wrap phase images, (**c**,**g**) were unwrap phase images, (**d**,**h**) were disparity images based on phase similarity.

Due to the resolution and frame rate of the light field camera, fringes with frequencies of 15, 12, and 10 were suitable and used in our experiment. We removed the calibration work for the projector to make the experimental procedure more flexible. The horizontal and vertical fringes were used to different objects separately to prove the applicability of the proposed method, and the experimental results were shown in Figure 9. The sine fringes were projected onto the object surface and the images modulated by the surface information captured by the light field camera were shown in Figure 9a,e. Subsequently, a four-step shifted phase method was used to calculate the captured structured light field data, and the resulting wrap phase images were shown in Figure 9b,f. The multi-frequency heterodyne method was used to unwrap the phase in Figure 9c,g. On the basis of the structured light field, EPI technology was used to process light field data, and the phase-similarity-based method was used instead of the structure tensor to calculate the slope of a straight line in EPI. Phase encoding ensured that there was a unique determination relationship between the phase value and the pixel in the scene, the morphological processing method was used to denoise and optimize the disparity map to improve its accuracy. After that, the disparity maps were obtained and shown in Figure 9d,h by using the proposed method based on phase similarity. The depth information of the measured object could be obtained by substituting the accurate disparity map based on phase similarity into the calibrated linear equation.

**Figure 10:**
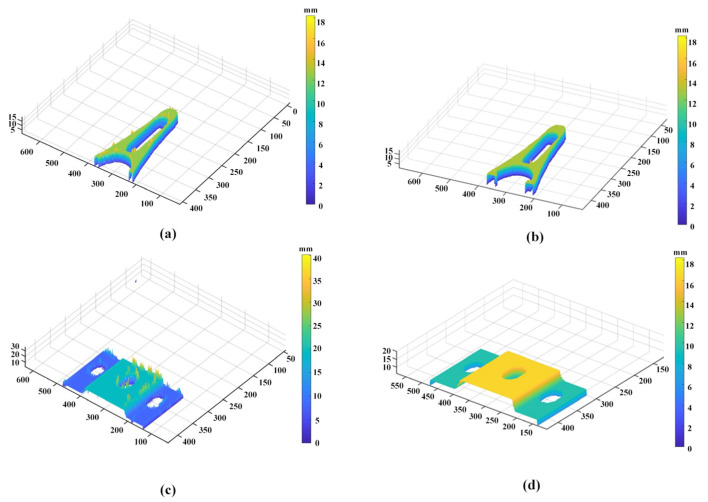
3D reconstruction results of different methods. (**a**) traditional EPI for object 1; (**b**) our approach for object 1; (**c**) traditional EPI for object 2; (**d**) our approach for object 2.

In our experiments, two different objects were used as the measured objects, and the reconstruction results were shown in Figure 10. It was obvious that the traditional EPI method produced aliasing and noise phenomenon in areas with similar textures, which led to large anomalies as shown in Figure 10a,c. Figure 10b,d showed the 3D reconstruction results of our proposed method. It is obvious from the results that the reconstruction result of our method can not only maintain the integrity but also have a smooth surface and without unnecessary noise or burr, which reflected the high accuracy of our method. In addition, the standard deviation of the reconstruction results was 0.3179 mm and 0.3865 mm compared with the ground truth of the measured objects, respectively. The comparative analysis results are presented in Table 1. Therefore, the experimental results demonstrated the feasibility and reliability of our proposed method.

## 6. Discussion and Conclusions

In this paper, a novel 3D reconstruction method of light field based on phase similarity is proposed to increase the accuracy of depth estimation and the applicability of EPI. In the calibration part, system calibration has been removed, and only the light field camera needs to be calibrated, which improves the simplicity of the operation. The linear relationship between disparity and depth can be obtained by acquiring the information of the feature points of the chessboard based on the imaging model and calibration method by using the light field camera. The calibration results and the relationship had been verified each other, and it not only can reflect the mapping relationship between disparity and depth in the scene but also deduces some parameter information of the light field camera, such as the distance *v*, the distance *b* between MLA and sensor, according to the formula deduced from the calibration. Then, the object is encoded by sinusoidal fringes, and accurate disparity maps can be calculated from scene phase information. Morphological processing is used for optimization and denoising subsequently. Therefore, it is obvious that 3D reconstruction of light field based on phase similarity can be realized by combining the calibrated linear relation and the accurate disparity map. Due to the uniqueness and accuracy of the phase information, it is obvious that the phase similarity in our method is more accurate than the structure tensor in the traditional EPI method. However, although this method has good performance in the situation of a single texture or similar texture scene. For some objects with complex morphology or small size, such as portrait sculptures, small workpieces, and so on, it is difficult to achieve accurate reconstruction due to the small difference between the modulated phase information and the original phase value. Hence, future work needs to focus on improving the resolution of the light field camera so that it can obtain more subtle phase changes to improve the applicability of the algorithm.

## Figures and Tables

**Table 1:** Comparative analysis of results.

Methods	Data (mm)		Error (mm)	
	Object 1	Object 2	Object 1	Object 2
Ground Truth	12	17		
Tradition EPI	12.9422	18.2741	0.9422	1.2741
Our approach	12.3179	17.3865	0.3179	0.3865

## Data Availability

Data available on request due to restrictions e.g., privacy or ethical. The data presented in this study are available on request from the corresponding author. The data are not publicly available due to privacy.

## References

[1] Lueke J.P., Rosa F., Marichal-Hernandez J.G., Sanluis J.C., Dominguez Conde C., Rodriguez-Ramos J.M. (2015). Depth from light fields analyzing 4d local structure. J. Disp. Technol..

[2] Wu G., Masia B., Jarabo A., Zhang Y., Wang L., Dai Q., Chai T., Liu Y. (2017). Light field image processing: An overview. IEEE J. Sel. Top. Sign. Proces..

[3] Sun J., Hossain M., Xu C., Zhang B. (2018). Investigation of flame radiation sampling and temperature measurement through light field camera. Int. J. Heat Mass Tran..

[4] Conde C.D., Luke J.P., Gonzalez F.R. (2019). Implementation of a Depth from Light Field Algorithm on FPGA. Sensors.

[5] Zhou W., Wei X., Yan Y., Wang W., Lin L. (2019). A hybrid learning of multimodal cues for light field depth estimation. Digit. Signal. Process..

[6] Rogge S., Schiopu I., Munteanu A. (2020). Depth Estimation for Light-Field Images Using Stereo Matching and Convolutional Neural Networks. Sensors.

[7] Liyanage N., Wijenayake C., Edussooriya C., Madanayake A., Agathoklis P., Bruton L., Ambikairajah E. (2020). Multi-depth filtering and occlusion suppression in 4-D light fields: Algorithms and architectures. IEEE Signal Proc. Mag..

[8] Pei R., Geng Z., Zhang Z., Cao X., Wang R. (2016). A novel optimization method for lenticular 3-D display based on light field decomposition. J. Disp. Technol..

[9] Chen N., Zuo C., Lam E.Y., Lee B. (2018). 3D Imaging Based on Depth Measurement Technologies. Sensors.

[10] Yu Z., Guo X., Ling H., Lumsdaine A., Yu J. Line assisted light field triangulation and stereo matching. Proceedings of the 2013 IEEE International Conference on Computer Vision.

[11] Heber S., Pock T. (2014). Shape from light field meets robust PCA. Proc. Eur. Conf. Comput. Vis..

[12] Wang T., Efros A.A., Ramamoorthi R. (2016). Depth estimation with occlusion modeling using light-field cameras. IEEE Trans. Pattern Anal..

[13] Tao M.W., Hadap S., Malik J., Ramamoorthi R. Depth from combining defocus and correspondence using light-field cameras. Proceedings of the 2013 IEEE International Conference on Computer Vision.

[14] Tao M.W., Srinivasan P.P., Hadap S., Rusinkiewicz S., Malik J., Ramamoorthi R. (2017). Shape estimation from shading, defocus, and correspondence using light-field, angular coherence. IEEE Trans. Pattern Anal..

[15] Wang T., Chandraker M., Efros A.A., Ramamoorthi R. SVBRDF-Invariant shape and reflectance estimation from light-field cameras. Proceedings of the 2016 IEEE Conference on Computer Vision and Pattern Recognition (CVPR).

[16] Wanner S., Goldluecke B. (2014). Variational light field analysis for disparity estimation and super-resolution. IEEE Trans. Pattern Anal..

[17] Kim C., Zimmer H., Pritch Y., Sorkine-Hornung A., Gross M. (2013). Scene reconstruction from high spatio-angular resolution light fields. ACM Trans. Graph..

[18] Suzuki T., Takahashi K., Fujii T. (2017). Sheared EPI analysis for disparity estimation from light fields. IEICE Trans. Inf. Syst..

[19] Zhou M., Su X., Chen W., You Z., Lu M., Jing H. (2014). Modulation measuring profilometry with auto-synchronous phase shifting and vertical scanning. Opt. Express..

[20] Zhou P., Zhang Y., Yu Y., Cai W., Zhou G. (2020). 3D shape measurement based on structured light field imaging. Math. Biosci. Eng..

[21] Duan H., Mei L., Wang J., Song L., Liu N. (2019). A new imaging model of Lytro light field camera and its calibration. Neurocomputing..

[22] Liu Z., Wu Q., Chen X., Yin Y. (2016). High-accuracy calibration of low-cost camera using image disturbance factor. Opt. Express..

[23] Jiang W., Wang Z. (2016). Calibration of visual model for space manipulator with a hybrid LM–GA algorithm. Mech. Syst. Signal. Process..

[24] Zhou P., Cai W., Yu Y., Zhang Y., Zhou G. (2019). A two-step calibration method of lenslet-based light field cameras. Opt. Laser Eng..

[25] Yao T., Sang X., Chen D., Wang P., Wang H., Yang S. (2019). Multi-view acquisition for 3D light field display based on external mask and compressive sensing. Opt. Commun..

[26] Wang S., Liu B., Chen Z., Li H., Jiang S. (2020). The Segmentation Method of Target Point Cloud for Polarization-Modulated 3D Imaging. Sensors.

[27] Dansereau D.G., Pizarro O., Williams S.B. Decoding, calibration and rectification for lenselet-based plenoptic cameras. Proceedings of the 2013 IEEE Conference on Computer Vision and Pattern Recognition.

